# Association of alpha-actinin-3 genotype with muscle mass and physical function in community-dwelling older adults

**DOI:** 10.1007/s41999-024-01080-0

**Published:** 2024-10-18

**Authors:** Daijo Shiratsuchi, Yoshiaki Taniguchi, Yuto Kiuchi, Shoma Akaida, Hyuma Makizako

**Affiliations:** 1 Graduate School of Health Sciences, Kagoshima University, 8-35-1 Sakuragaoka, Kagoshima, 890-8544 Japan; 2 Department of Physical Therapy, School of Health Sciences, Faculty of Medicine, Kagoshima University, 8-35-1 Sakuragaoka, Kagoshima, 890-8544 Japan; 3 Department of Rehabilitation, Faculty of Nursing and Welfare, Kyushu University of Nursing and Social Welfare, 888 Tominoo, Tamana, Kumamoto 865-0062 Japan; 4 Department of Preventive Gerontology, Center for Gerontology and Social Science, National Center for Geriatrics and Gerontology, 7-430 Morioka, Obu, Aichi 474-8511 Japan

**Keywords:** Body composition, Candidate gene, Older people, Single-nucleotide polymorphism

## Abstract

**Purpose:**

Polymorphisms (rs1815739; R577X) in the gene encoding alpha-actinin-3 (ACTN3) are thought to be associated with body composition and physical function in older people and athletes. RR homozygotes are associated with greater expression of ACTN3 protein in muscle than the X-allele carriers. We aimed to investigate the association between *ACTN3* R577X polymorphism and appendicular skeletal muscle mass, walking speed, and muscle strength in older adults.

**Methods:**

A cross-sectional analysis was performed on 265 community-dwelling older adults (mean age 74.0 ± 5.8 years, 63.4% female) who provided data on *ACTN3* gene polymorphisms and completed surveys in the Tarumizu study conducted between 2018 and 2019. Genetic polymorphisms were categorized as RR homozygous and X allele. Muscle mass was assessed using the appendicular skeletal muscle mass index (ASMI), and physical function was assessed based on walking speed and relative muscle strength. Those in the bottom 25% for each sex were considered “low” and the association with *ACTN3* genotype was examined.

**Results:**

Considering *ACTN3* polymorphism, 72 participants were RR homozygotes (27.2%) and 193 were X-allele carriers (72.8%). After adjusting for potential confounders, RR homozygosity was associated with not having low muscle mass (odds ratio 0.39, 95% confidence interval 0.19–0.82, *p* = 0.013) but not with low walking speed and muscle strength.

**Conclusion:**

The association between *ACTN3* genotype and physical function in community-dwelling older adults is not clear; however, it is considered to be associated with muscle mass.

## Key summary points

**Aim:**

To investigate the association between ACTN3 R577X polymorphism and appendicular skeletal muscle mass, walking speed, and muscle strength in older adults.

**Findings:**

RR homozygosity in the ACTN3 gene was associated with a lower likelihood of having low muscle mass. However, no significant association existed between ACTN3 polymorphism and walking speed or muscle strength.

**Message:**

ACTN3 R577X polymorphism may be associated with muscle mass but not physical function in terms of walking speed and muscle strength in older adults.

## Introduction

Alpha-actinin-3 (ACTN3) is a protein expressed only in fast muscle fibers and forms a significant component of the contractile apparatus in the Z-line, where it cross-links and anchors actin filaments [1, 2]. Mutation in the codon for amino acid 577 in exon 16 of *ACTN3* (rs1815739; R577X) results in changing amino acid arginine (R) to a stop codon (X). Individuals with the X allele show inadequate expression of the ACTN3 protein in muscles compared to that in individuals with the RR homozygotes [3]. *ACTN3* is absent in approximately 18% of the population globally and in 25% of the population at high latitudes [4, 5]. To date, it has not been confirmed that *ACTN3* gene polymorphism causes any muscle disease, although it has been suggested that it may affect the muscle phenotype [6, 7]. Indeed, meta-analyses have shown that the R allele is common in power athletes, such as sprinters or weightlifters. [7]. Previous studies have reported that the cross-sectional area between type II fibers is greater in those with RR than in those with XX, whereas the composition and proportion of muscle fibers remain unaffected [8, 9].

Physical function and muscle mass in old age are thought to be genetically influenced; however, their association with the *ACTN3* genotype remains controversial. Although previous studies on community-dwelling older adults have not examined this association thoroughly, a few studies have reported that the X allele is associated with an increased risk of falls in older women and reduced grip strength and walking speed in older men [10, 11]. In addition, a previous study found an association between XX homozygotes and reduced muscle mass, as measured by dual-energy X-ray absorptiometry [12], which may have an impact on body composition and physical function in older adults. However, some reports focusing on *ACTN3* gene polymorphisms in community-dwelling older adults found no association between muscle mass and physical function [13, 14], suggesting the need for careful interpretation and further research.

Maintaining good physical function and muscle mass is essential for good health in old age, and for preventing diseases and nursing care [15]. Genetic information can potentially be used for various aspects, such as early detection, prognosis prediction, and choice of treatment strategy [16, 17]. If there is an association between *ACTN3* polymorphisms and muscle mass and physical function, we speculate that genetic polymorphisms could be used to determine the likelihood of a future decline in body composition and physical function. Therefore, this study aimed to determine the association between *ACTN3* gene polymorphisms and muscle mass, muscle strength, and walking speed in community-dwelling older adults. We hypothesized that the RR homozygote is associated with not having low skeletal muscle mass, walking speed, and muscle strength.

## Methods

### Study design and participants

This study included 283 older adults who participated in a regional cohort study (Tarumizu Study 2018, 2019) and underwent genetic testing. The Tarumizu Study is a cohort of community-based health examinations conducted in Tarumizu, Japan, from June to December 2018 and June to December 2019 [18]. Participants were excluded if they required assistance or care (*n* = 1), had a history of stroke (*n* = 8), or had missing data (*n* = 9). A cross-sectional analysis was performed with 265 participants (mean age 74.0 ± 5.8 years; 97 males, 168 females) in the final analysis. This study was performed in accordance with the Declaration of Helsinki and approved by the Institutional Review Board of the University of the Ethics Committee on Epidemiological and Related Studies, Sakuragaoka Campus, Kagoshima University (ethics approval code. no. 170351, 190319). Prior written informed consent was obtained from all the participants.

### *ACTN3* genotype

The *ACTN3* genotype was obtained from buccal mucosa samples of each participant [19]. This was determined using a DNA Exercise Genetic Testing Kit (Hersires International, Hiroshima, Japan). After ensuring that the participants had not eaten or consumed any fluids 30 min prior to testing, they were requested to gargle with water. Samples were collected by rubbing the inside of the right and left cheeks for one minute using sterile cotton swabs (Tomy Works, Sakai, Japan). The Mag Max DNA Multi-Sample Ultra Kit (Thermo Fisher Scientific, Paisley, UK) and King Fisher Flex Purification System (Thermo Fisher Scientific, Paisley, UK) were used to extract genomic DNA from the buccal mucosa in the swabs. The *ACTN3* genotype (rs1815739; R577X) was analyzed using polymerase chain reaction (PCR) with two sets of primers (PCR-CTPP) using the KAPA2G Robust PCR Kit (Kapa Biosystems, Wilmington, MA, USA). Carriers of the X allele were classified as RR and RX/XX, because they showed similar phenotypic responses [20, 21].

### Muscle mass and physical function

Skeletal muscle mass was assessed using a multi-frequency bioelectrical impedance analyzer (InBody 470; InBody Japan, Tokyo, Japan) [22]. Appendicular skeletal muscle mass (ASM) was calculated as the sum of muscle masses of the limbs, and the ASM index (ASMI, kg/m^2^) was then calculated using the formula: ASMI = ASM (kg)/height (m)^2^.

Walking speed was measured by trained raters (all physiotherapists) using a standardized procedure for the 10 m walk test [23]. The walking course of 14 m in a hallway consisted of 2 m for warm-up, 10 m for speed measurement, and 2 m for slowing down to a stop. Walking speed was measured using an infrared sensor (YW; Yagami Inc., Aichi, Japan) placed in the middle 10 m of the 14 m straight walkway. Instructions were provided to the participant to “walk at a comfortable pace”. Grip strength was determined by measuring the maximum grip strength of the participant’s dominant hand using a Smedley handheld dynamometer (Grip-D; Takei Ltd., Niigata, Japan) [24]. The test was performed once in the standing position [25]. Relative muscle strength (grip strength [kg]/body weight [kg]) was calculated to reduce the effect of body size on grip strength [26].

### Assessment of characteristics

Demographic information included age (years), sex, education (years), body mass index (BMI), medication (n/day), history of hypertension, hyperlipidemia, osteoporosis, diabetes, and ischemic heart disease (including angina and myocardial infarction), living alone status, and exercise habits (at least twice a week).

### Statistical analysis

Data are presented as mean ± standard deviation for continuous variables and as numbers and percentages for nominal variables. Differences in RR homozygotes and X-allele carriers were analyzed using Student’s *t* test and *x*^2^ test. Referring to previous studies that considered the distribution and defined cut-off values [27, 28], a decline was defined as being below the first quartile for ASMI, walking speed, and relative muscle strength by sex. For each variable, low muscle mass was ≤ 6.8 kg/m^2^ for males and ≤ 5.4 kg/m^2^ for females; low walking speed was ≤ 1.2 m/s for both sexes; and low muscle strength was ≤ 0.46 kg/kg for males and ≤ 0.34 kg/kg for females. Multivariate logistic regression analysis was used to examine the association between *ACTN3* genotype and low muscle mass, walking speed, and muscle strength. As covariates, age, sex, education, medication, living alone, and exercise habits were included; in addition, walking speed and muscle strength were included if the dependent variable was low muscle mass, and ASMI if the walking speed or muscle strength was low. Statistical significance was set at *p* < 0.05, and adjusted odds ratios (OR) and 95% confidence intervals (CI) were calculated. All analyses were performed using IBM SPSS Statistics 28.0 (IBM Japan, Tokyo, Japan).

## Results

### Characteristics of participants

Table 1 presents the characteristics of the participants. Of the total participants, 168 (63.4%) were female with less education (*p* = 0.037), lower ASMI (*p* < 0.001), and lower muscle strength (*p* < 0.001) than males. More women had hyperlipidemia (*p* = 0.005), osteoporosis (*p* < 0.001), and were living alone (*p* = 0.006), while fewer women had diabetes (*p* = 0.030).

**Table 1:** Characteristics of the participants

	Overall (*n* = 265)	Male (*n* = 97)	Female (*n* = 168)	*p* value
Age, years, mean ± SD	74.0 ± 5.8	74.2 ± 5.8	73.9 ± 5.8	0.707
Education, years, mean ± SD	11.7 ± 2.3	12.1 ± 2.7	11.4 ± 1.9	**0.037**
BMI, kg/m^2^, mean ± SD	23.0 ± 3.2	23.2 ± 2.8	22.9 ± 3.4	0.419
Medication, number/day, mean ± SD	3.0 ± 3.0	3.1 ± 3.1	3.0 ± 3.0	0.806
Chronic disease, number (%)				
Hypertension	110 (41.5)	47 (48.5)	63 (37.5)	0.081
Hyperlipidemia	64 (24.2)	14 (14.4)	50 (29.8)	**0.005**
Osteoporosis	53 (20.0)	1 (1.0)	52 (31.0)	** < 0.001**
Diabetes	36 (13.6)	19 (19.6)	17 (10.1)	**0.030**
Ischemic heart disease	21 (7.9)	10 (10.3)	11 (6.5)	0.275
Living alone, number (%)	76 (28.7)	18 (18.6)	58 (34.5)	**0.006**
Exercise habit, number (%)	185 (69.8)	68 (70.1)	117 (69.8)	0.937
Muscle mass, mean ± SD				
ASMI, kg/m^2^	6.3 ± 1.0	7.2 ± 0.6	5.8 ± 0.7	** < 0.001**
Physical function, mean ± SD				
Walking speed, m/s	1.3 ± 0.2	1.3 ± 0.2	1.3 ± 0.2	0.623
Grip strength, kg	25.2 ± 7.7	33.0 ± 5.9	20.6 ± 4.1	** < 0.001**
Relative muscle strength, kg/kg	0.46 ± 0.11	0.54 ± 0.09	0.41 ± 0.09	** < 0.001**

Significant *p* values are indicated in bold

*ASMI* appendicular skeletal muscle mass index, *BMI* body mass index

### Comparison of characteristics among those with the* ACTN3* genotype

The RR homozygotes were present in 72 (27.2%) participants; they were less likely to be female (*p* = 0.013), took less medication (*p* = 0.015), and had less osteoporosis (*p* = 0.027) (Table 2). Table 3 shows the comparison of muscle mass and physical function by sex for the ACTN3 genotype. In males, RR homozygotes had higher grip strength than X-allele carriers (*p* = 0.049).

**Table 2:** Comparison of characteristics among *ACTN3* genotype

	RR homozygotes (*n* = 72)	X-allele carriers (*n* = 193)	*p* value
Age, years, mean ± SD	73.1 ± 4.7	74.3 ± 6.2	0.097
Sex, female, number (%)	37 (51.4)	131 (67.9)	**0.013**
Education, years, mean ± SD	11.8 ± 2.2	11.6 ± 2.3	0.586
BMI, kg/m^2^, mean ± SD	23.3 ± 2.7	22.9 ± 3.3	0.344
Medication, number/day, mean ± SD	2.4 ± 2.5	3.3 ± 3.1	**0.015**
Chronic disease, number (%)			
Hypertension	26 (36.1)	84 (43.5)	0.276
Hyperlipidemia	23 (31.9)	41 (21.2)	0.070
Osteoporosis	8 (11.1)	45 (23.3)	**0.027**
Diabetes	9 (12.5)	27 (14.0)	0.753
Ischemic heart disease	6 (8.3)	15 (7.8)	0.880
Living alone, number (%)	17 (23.6)	59 (30.6)	0.265
Exercise habit, number (%)	51 (70.8)	134 (69.4)	0.825

Significant *p* values are indicated in bold

*BMI* body mass index

**Table 3:** Comparison of muscle mass and physical function by sex for ACTN3 genotype

	RR homozygotes	X-allele carriers	*p* value
Male (n = 97)			
Muscle mass, mean ± SD			
ASMI, kg/m^2^	7.3 ± 0.6	7.1 ± 0.7	0.172
Physical function, mean ± SD			
Walking speed, m/s	1.3 ± 0.2	1.3 ± 0.2	0.837
Grip strength, kg	34.6 ± 5.9	32.1 ± 5.8	**0.049**
Relative muscle strength, kg/kg	0.56 ± 0.08	0.53 ± 0.10	0.103
Female (n = 168)			
Muscle mass, mean ± SD			
ASMI, kg/m^2^	5.9 ± 0.6	5.8 ± 0.7	0.496
Physical function, mean ± SD			
Walking speed, m/s	1.3 ± 0.2	1.3 ± 0.2	0.612
Grip strength, kg	21.1 ± 4.1	20.5 ± 4.1	0.480
Relative muscle strength, kg/kg	0.41 ± 0.08	0.41 ± 0.09	0.824

Significant *p* values are indicated in bold

*ASMI* appendicular skeletal muscle mass index

### Rates of low muscle mass, walking speed, and muscle strength in *ACTN3* genotype

**Fig. 1:**
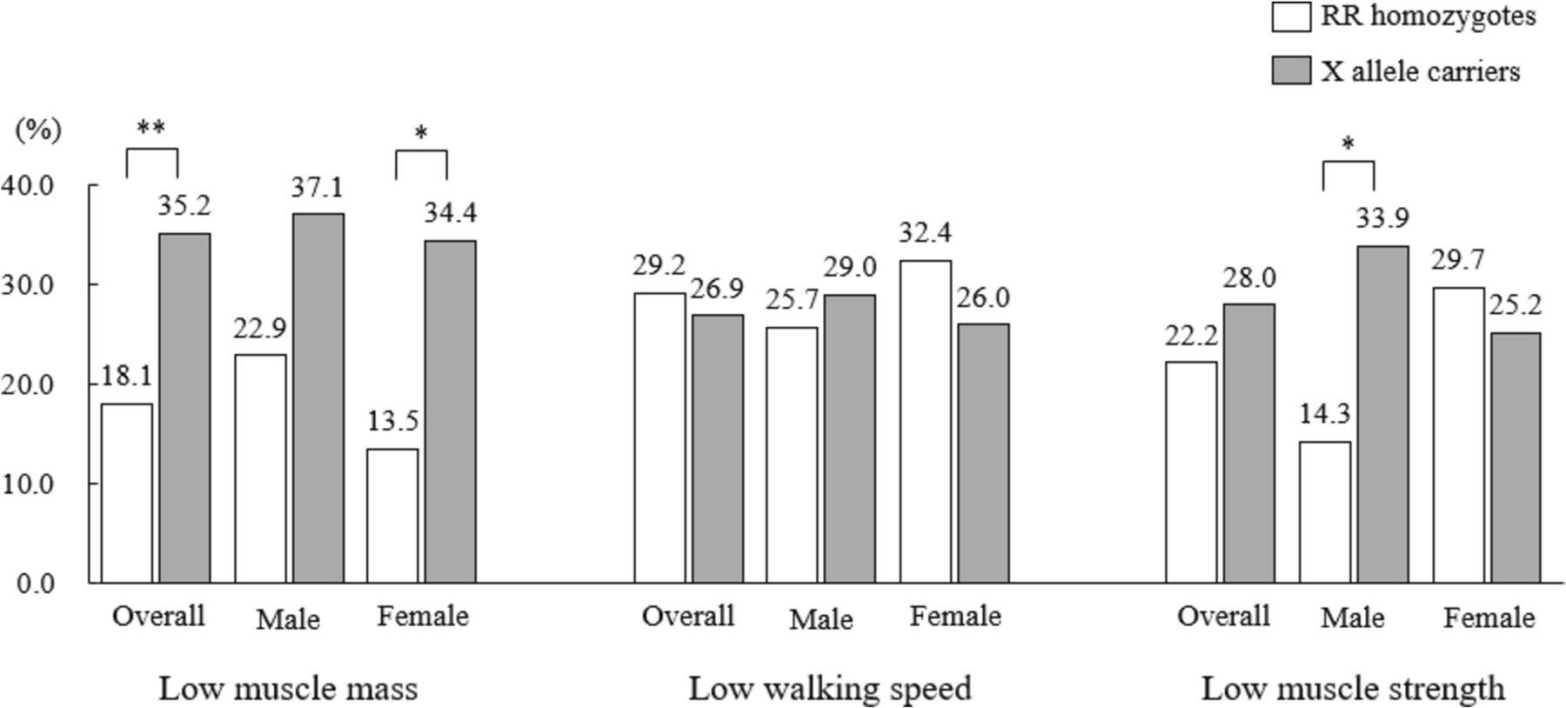
Rates of low muscle mass, low walking speed, and low muscle strength in *ACTN3* genotype **p < 0.01, *p < 0.05

The proportions of low muscle mass, walking speed, and muscle strength for each *ACTN3* genotype are shown in Fig. 1. A significantly lower proportion of RR homozygotes had low muscle mass compared to X-allele carriers (RR homozygotes: 18.1%, X-allele carriers: 35.2%, *p* = 0.007). Similarly, the rate of low muscle mass was significantly lower in females with RR homozygotes (RR homozygotes: 13.5%, X-allele carriers: 34.4%, *p* = 0.014). Regarding low walking speed, there were no significant differences between RR homozygotes and X-allele carriers across the overall population, as well as within male and female subgroups. In terms of muscle strength, a significantly lower proportion of males with RR homozygotes exhibited low muscle strength compared to those with the X allele (RR homozygotes: 14.3%, X-allele carriers: 33.9%, *p* = 0.036).

### Association of* ACTN3* genotype with muscle mass and physical function

Even after adjustment for potential covariates, the presence of RR homozygotes was significantly associated with no loss of muscle mass (OR 0.39, 95% CI 0.19–0.82, *p* = 0.013). No association was found between *ACTN3* genotype and low walking speed (OR 1.55, 95% CI 0.81–2.98, *p* = 0.188) and low muscle strength (OR 0.92, 95% CI 0.46–1.84, *p* = 0.821) (Table 4).

**Table 4:** Association between *ACTN3* genotype with muscle mass and physical function

	Low muscle mass OR (95% CI)^1^	Low walking speed OR (95% CI)^2^	Low muscle strength OR (95% CI)^2^
*ACTN3* genotype			
RR homozygotes	0.39 (0.19–0.82)*	1.55 (0.81–2.98)	0.92 (0.46–1.84)
X-allele carriers	Reference	Reference	Reference

*OR* odds ratio, *CI* confidence interval. Low muscle mass: below 6.8 kg/m^2^ for males and 5.4 kg/m^2^ for females; Low walking speed: below 1.2 m/s for both sexes; Low muscle strength: below 0.46 kg/kg for males and 0.34 kg/kg for females

^1^Adjusted model: adjusted for age, sex, education, medications, living alone, exercise habits, walking speed, and relative muscle strength

^2^Adjusted model: adjusted for age, sex, education, medications, living alone, exercise habits, and ASMI

*p < 0.05

## Discussion

This study aimed to determine the association between *ACTN3* genotype and skeletal muscle mass, walking speed, and muscle strength in community-dwelling older adults. The results showed that RR homozygosity was not associated with the low ASMI category.

Genetic research has mainly focused on Western populations [29, 30], with few reports on Asian populations. In our study, 27.2% of the participants were RR homozygotes, a proportion consistent with the previous findings [10, 12]. Because genes are influenced by each other and the environment, including lifestyle [31, 32], it is necessary to examine their relationships in countries with different races and cultures. The *ACTN3* genotype is thought to have some protective effect against the loss of muscle mass, function, and bone mineral density in older people [18, 33]. A longitudinal study of *ACTN3* gene polymorphisms, physical function, and body composition in older adults showed that women homozygous for XX were more likely to develop lower limb limitations [34]. Studies on Korean and Japanese participants revealed an association between *ACTN3* genotype and skeletal muscle mass [12, 35], which is consistent with the findings of the present study. In contrast, the previous studies in Caucasians have not found an association with skeletal muscle mass, which may require further validation owing to the relatively small sample size [14, 36, 37].

Previous studies have reported an association between *ACTN3* polymorphisms and physical function. Older adults with XX homozygotes have lower grip strength, walking speed, and poorer chair stand test results than those with RR homozygotes [11, 38]. Our univariate analysis also showed that grip strength was significantly higher in RR homozygotes in males. However, this association did not hold in the logistic regression analysis after adjusting for covariates. It is well known that grip strength varies not only by sex and age but also by anthropometric characteristics. However, studies suggest that the association between grip strength and health outcomes remains consistent regardless of how relative values are calculated—whether relative to height, weight, fat-free mass, BMI, or fat-free mass index [39]. Therefore, in this study, we focused on examining the association with relative muscle strength, calculated by dividing grip strength by body weight. In general, there is a proportional relationship between muscle mass and muscle strength, but this association is not always decisive in old age. Prior studies have reported that declines in muscle strength occur in older adults regardless of changes in muscle mass [40], suggesting that the factors associated with muscle mass may differ from those associated with muscle strength. Our findings indicate that the ACTN3 genotype may be more closely related to muscle mass than strength. One of the characteristics of fast muscle fibers is that they are thicker than slow muscle fibers [41]. It is assumed that *ACTN3* is RR homozygous, which means that the absolute muscle volume is higher than that of X-allele carriers, who have a smaller cross-sectional area of fast-twitch muscle fibers. It has also been reported that atrophy and regression of fast muscle fiber occur primarily as changes in skeletal muscles in old age [42]. RR homozygotes have a large cross-sectional area of fast-twitch muscle fibers, indicating that they have a larger absolute skeletal muscle mass and may be able to cope with age-related reductions in fast-twitch muscle fibers. Along with muscle mass, potential influences on muscle strength include reduced motor units, increased stimulus thresholds, and increased transmission instability at the neuromuscular junction [43, 44].

The care and protection of skeletal muscle mass is becoming increasingly important because of its association with mortality [15]. Although physical function and muscle mass decrease with aging, individual differences exist in the rate of decline [45]. However, the biological mechanisms underlying these differences have not yet been fully elucidated. Recent insights suggest that while muscle mass is a significant factor in sarcopenia outcomes, muscle quality increasingly appears critical [46]. Thus, although our findings link the ACTN3 genotype predominantly with muscle mass rather than physical performance, it does not diminish the importance of either factor in the broader context of aging and disability. In the present study, *ACTN3* gene polymorphism was not associated with muscle strength and walking speed. One possible explanation for these findings could be the relatively higher heritability of body composition parameters compared to those of physical function, which suggests that physical function may be influenced by a larger number of non-genetic factors [47–49]. For example, strength training has been reported to be equally effective in improving physical function across different ACTN3 genotypes [50, 51], underscoring the potential impact of environmental and lifestyle factors. Furthermore, our study adjusted for a more extensive range of covariates than previous studies, which might have contributed to the differences in observed associations. However, the association of *ACTN3* gene polymorphism with increased physical function remains open to investigation, as it has been reported that in older adults, *ACTN3* gene polymorphism is associated with muscle power such as knee extension torque [52]. Future studies should examine the association between physical functions that require fast and strong contractions, such as the chair stand test and maximum walking speed, and more robust and reliable tools for assessing physical function, such as the short physical performance battery (SPPB).

This study has several limitations. First, it was not possible to explain the causal relationship, because the analysis was cross-sectional. Second, the study population comprised older Japanese adults who were able to live in a community; thus, the results cannot be generalized to all older people. Third, considering the sample size, sub-analyses for sex or age were not performed. Fourth, there are unmeasured confounders, such as cognitive, emotional and other geriatric assessments, detailed comorbidities, and type of exercise and nutrient intake. Fifth, the number of drugs used by participants was recorded, but specific types of drugs were not heard. Finally, we set a cut-off value for the participants in this study and analyzed them. Although these points need to be considered carefully, we believe that this is a crucial study showing the potential association between X allele of *ACTN3* and age-related changes in muscle mass in old adults.

## Conclusion

*ACTN3* gene polymorphisms are associated with a low ASMI in community-dwelling older adults; however, their association with physical function is unclear. Future studies should ensure a sufficiently large sample size to allow for robust subgroup analyses by sex, verifying the causal relationship and clarifying whether environmental factors may reduce the influence of genes.

## Data Availability

The data underlying this article cannot be shared publicly due to privacy or ethical restrictions. The data will be shared on reasonable request to the corresponding author.
